# Association of FADS2 rs174575 gene polymorphism and insulin resistance in type 2 diabetes mellitus

**DOI:** 10.4314/ahs.v20i4.30

**Published:** 2020-12

**Authors:** Shilpa S Shetty, Kumari N Suchetha, Devi Harshini, KP Sharmila, Srinidhi Rai

**Affiliations:** 1 Central Research Laboratory, K.S. Hegde Medical Academy, Nitte (Deemed to be University); 2 Department of Biochemistry, K.S. Hegde Medical Academy, Nitte (Deemed to be University)

**Keywords:** Fatty acid desaturase, gene polymorphism, type 2 diabetes mellitus

## Abstract

**Background:**

Many risk factors contribute to the pathogenesis of diabetes. Gene and lifestyle factors are considered to be the major contributors. A dietary pattern is attributed to be one of the lifestyle risk factors favoring diabetes. The present study aims to find an association between fatty acid desaturase (FADS) gene polymorphism and glycemic profile in type 2 diabetes mellitus (T2DM).

**Methodology:**

A total of 429 subjects were included in the study on the basis of inclusion and exclusion criteria, of which 213 and 216 subjects were diabetic and control, respectively. Body mass index was calculated. Fasting plasma glucose, glycated hemoglobin (HbA1c) and insulin were measured using commercially available kits. rs174575 of FADS2 was selected based on previous publications and identified using the dbSNP database. To compare the biochemical parameters with the genotype, the following three models were used: additive model (CC vs CG vs GG), dominant model (CC + CG vs GG), and recessive model (CC vs CG + GG).

**Results and Discussion:**

FBS, HbA1c, insulin, HOMA-IR, and HOMA-B exhibited a high and statistically significant difference between subjects and controls. The three models exhibited a statistically significant difference between FBS, HOMA-IR, and HOMA- B (p<0.05).

**Conclusion:**

The distribution of rs174575 genotype differed significantly between the subjects and controls in the present study. The study revealed that genetic variation in FADS2 is an additional facet to consider while studying the risk factors of T2DM.

## Introduction

Insulin resistance (IR) is a precursor of type 2 diabetes mellitus (T2DM). Insulin resistance is multifaceted, and the etiology of insulin resistance is complicated with implications from several factors; thus, deciphering T2DM remains challenging. Improved understanding and the risk prediction of the pathogenesis of insulin resistance is crucial 1. Evidence suggests that oxidative stress; inflammation; and genetic, habitual, environmental, and epigenetic factors are involved 2. The epidemiological profile of T2DM in India greatly differs from that in the west. Despite low body mass index (BMI), Asian-Indians have a group of clinical and biochemical traits, which is referred to as the “Asian-Indian phenotype,” making them more susceptible to the development of T2DM 3; 80% of patients with T2DM in India are non-obese, and 1/4^th^ of T2DM in India are of BMI < 19 kg/m^2^. The dietary patterns of Indians are vastly different from a typical “western” diet, with relatively high carbohydrate intake and low fat intake. The main sources of fat are of plant origin rather than animal origin because the majority of population is vegetarian, resulting in a diet that is relatively low in saturated fatty acids (SFAs), high in n-6 polyunsaturated fatty acids (PUFAs), and very low in n-3 PUFAs. Though this appears as a good dietary composition as per global standards, the undeniable increase in the incidence of obesity, diabetes, and cardiovascular diseases in India requires immediate attention. Fats are the main sources of energy for the body, but the type and total amount of fat consumed daily play an important role in maintaining the state of health. The dietary fatty acids in the body undergo a series of enzyme catalyzed reaction to produce bioactive molecules. FADS is one of the major genes associated with the desaturation of FAs. D6D and D5D are encoded by FADS2 and FADS1, respectively, located on chromosome 11 4. Polymorphism in FADS has an especially strong association with the high plasma proportion of arachidonic acid that is known to be a precursor for bodily inflammation 5,6. The alteration in FA composition results in altered metabolic function, thereby increasing or decreasing the risk of disease because PUFA also acts as biological ligands to functional pathways. Several studies have reported that the desaturation pathway is highly important for glucose homeostasis in the human body. Our current knowledge of the impact of genetic polymorphisms and their potential role in the development of diseases is limited. Therefore, SNPs in FADS cluster should be identified and characterized, thereby providing a new sight to the role of fatty acid gene polymorphism in insulin resistance and T2DM and enabling the development new treatment regimens for the prevention and management of T2DM.

## Materials and Method

### Study Design and Population

This study was reviewed and approved for human subjects by the Central Ethics Committee of Nitte (Deemed to be University); Ref NU/CEC/PhD-16/2014 dated 9-10-2014. A total of 429 subjects were included in the study on the basis of inclusion and exclusion criteria; there were 213 (49.70%) subjects with T2DM (diabetic group) and 216 (50.30%) were controls (control group). The control group included 105 (48.60%) males and 111 (51.40%) females, whereas the diabetic group included 136 (63.80%) males and 77 (36.20%) females. Informed consent was duly signed by each subject.

### Inclusion and Exclusion Criteria

**Diabetic Group**

**Inclusion Criteria**

- Subjects with T2DM according to ADA criteria 6 diagnosed for at least 3 months prior to screening.
- Subjects in the age group of 30–60 years from both the sexes.

**Exclusion /Criteria**

- Pregnant women.

**Control Group**

**Inclusion Criteria**

- Subjects in the age group of 30–60 years of both sexes visiting for regular health check-ups.

**Exclusion Criteria**

- Pregnant women.

### Anthropometric Measurements

Bodyweight (kg) and height (cm) were measured. BMI was calculated as bodyweight (kg) divided by height in (m^2^) (kg/m^2^).

### Sample Collection and Biochemical Estimations

Five mL of fasting blood sample was collected for biochemical estimations, such as glucose, glycated hemoglobin (HbA1c), and insulin; FA analysis; and DNA isolation. Fasting plasma glucose was measured by the glucose oxidase method (LyphoCHEKTMAGAPPE). HbA1c was measured using ion exchange resin method on spectrophotometer (Nycocard READER, Abbott). Plasma insulin was measured using a commercially available human insulin kit (Accu- Bind, Monobind Inc., US).

### Calculation of HOMA-IR and HOMA-B

The HOMA of IR index (HOMA-IR) was calculated using the following formula 7:

HOMA-IR = Fasting glucose _(mmol/dl)_ × Fasting plasma insulin _(µIU/ml)_/22.5.

HOMA-B was calculated using the following formula 7:

HOMA-B= 20 × Fasting insulin (µIU/ml)/fasting glucose (mmol/ml) − 3.5.

### DNA Isolation and Quantification

The isolation of DNA from whole blood was performed using a standardized protocol by Sergeant et al., 2012 8. The concentration of DNA per sample was determined using bio-spectrophotometer and stored at −20°C until further analysis. The integrity of the genomic DNA was examined on 0.8% agarose gel. DNA in the gel was visualized using gel documentation system (Biorad). The DNA was quantified by measuring optical density at 260 nm and 280 nm in a bio-spectrophotometer. The ratio of 260/280nm was calculated for measuring the purity of DNA sample, and the DNA samples for which the ratio was 1.7–1.9 was stored at −20°C until further analysis.

### SNP Selection

The SNP rs174575 of FADS2 was selected according to previous publications 9–12 and identified using the dbSNP database 13 based on the National Centre for Biotechnology Information (NCBI) 12. The primer pair for SNP rs174575 (designed for the study) involved in the study was as follows: Forward: AGGCAGATGGACCTGGATTTGA and Reverse TGGCTTGCAAATAGACTCATCTCC. PCR conditions included initial desaturation at 95°C for 3 min, followed by 95°C for 1 min, 58°C for 30 sec, 72°C for 1 min for 35 cycles, final extension at 72°C for 10 min and holding at 4°C. The amplified products were purified using PCR-clean up kit (Sigma-Aldrich); the products were sequenced using Sanger sequencing method at Eurofins Genomics India Pvt. Ltd. Bengaluru, Karnataka, India.

## Statistical Analysis

Statistical analyses were performed using SPSS (version 20.0, IBM, Armonk, NY, USA). The Kolmogorov-Smirnov test was used to assess the normality of variables. Normal quantitative variables were reported as mean ± SD. To compare clinical characteristics, student's t-test and K-independent nonparametric analysis (Mann-Whitney U test) were used for the normal and non-normal distribution, respectively. Pearson's coefficient was used to associate some of the normal variables with each other in all subjects. One-way analysis of variance and Bonferroni correction tests were used to compare the variables between genotypes in each group.

## Results

### General Characteristics of the Study Population

Table 1 depicts the anthropometric parameters of the study population. Age and weight showed statistically significant difference between the two groups.

**Table 1:** Anthropometric Parameters of Study Population

	Control (N = 216) (mean±SD)	Diabetic (N = 213) (mean±SD)	p-value
**Age (yrs)**	47.44± 10.16	51.03 ± 8.25	<0.001
**Weight (Kg)**	60.15 ± 12.06	62.94 ± 10.32	0.011
**Height (Cm)**	158.98 ± 13.98	160.6 ± 14.18	0.23
**BMI (Kg/m^2^)**	23.961 ± 4.749	24.395 ± 4.018	0.308

^✤^ P-value = 0.05 was considered statistically significant.

*Student t-test: data are shown as mean ± SD

### Comparison of Glycemic Profile between the two Groups

The mean FBS (mg/dL), HbA1c (%), and insulin (pmol/L) levels in the control group were lower than those in the diabetic group. FBS, HbA1c, and insulin showed a high and statistically significant difference between the two groups (p < 0.001).

Additionally, HOMA-IR and HOMA-B exhibited a statistically significant difference between the two groups (Table 2).

**Table 2:** Comparison of Glycemic Profile between the Control and Diabetic Groups

Parameters	Control (N = 216) (mean±SD)	Diabetic (N = 213) (mean±SD)	p-value
FBS (mg/dL)	97.41 ± 14.04	192.77 ± 68.19	<0.001*
HbA1c (%)	4.63 ± 0.82	6.88 ± 1.92	<0.001*
Insulin (pmol/L)	30.59± 8.45	41.17 ± 3.37	<0.001*
HOMA-IR✥	1.007 (0.865–1.208)	2.57 (2.016–3.314)	<0.001
HOMA-B✥	45.633 (32.98–63.139)	19.037 (13.066–28.606)	<0.001

^✤^ P-value ≤ 0.05 was considered statistically significant.

* Student t-test: data are shown as mean ± SD.

✥ Mann–Whitney U test: data are shown as median (interquartile range).

Allele and Genotype Frequencies for FADS 2 rs174575 CC genotype was wild type homozygote genotype for rs174575 C>G, while GG was homozygous recessive variant genotype. CG was heterozygous genotype for rs174575. The allele frequency of C and G was 0.96 (96%) and 0.04 (4%), respectively, in the control group; in the diabetic group, is the frequency was 0.91 (91%) and 0.09 (9%), respectively. For the control and diabetic groups, the genotype frequency of CC, CG, and GG was 0.84 (95%), 0.1472 (4%), and 0.0064 (1%) and 0.807 (84%), 0.1770 (14%), and 0.0144 (2%), respectively. SNP is in Hardy Weinberg Equilibrium (HWE)-P= 1.732 (P > 0.05).

### Comparison of the Glycemic Profile Composition between the Diabetic subjects Based on Genotype

To compare glycemic profile with the genotype, the following three models were used in this study: additive model (CC vs CG vs GG), dominant model (CC + CG vs GG), and recessive model (CC vs CG + GG). In the additive model (CC vs CG vs GG), a statistically significant difference was observed in FBS, HOMA-IR and HOMA- B between three genotypes with a p-value of 0.022, 0.043 and 0.018, respectively. In the dominant model (CC+CG vs GG), a statistically significant difference was observed in FBS, HOMA-IR and HOMA- B between three genotypes with a p-value of 0.010, 0.026 and 0.016 respectively. In the recessive model (CC vs CG+GG) statistically significant difference was observed in HOMA- B (p=0.027), HOMA-IR showed a p-value of 0.042 (Table 3).

**Table 3:** Comparison of Glycemic Profile between the Diabetic Individuals Using Different Genotype Models

	Additive Model (CC vs CG vs GG) (mean±SD)				Dominant Model (CC + CG vs GG) (mean±SD)			Recessive Model (CC vs CG + GG) (mean±SD)		
Parameters	CC (N=180)	CG (N=29)	GG (N=4)	p-value	CC + CG (N=209)	GG (N=4)	p-value	CC (N=180)	CG + GG (N=33)	p-value
**FBS**	189.19 ± 5.05	202.88 ± 12.66	280.33 ± 33.87	0.022	191.10 ± 4.68	280.24 ± 33.87	0.010	189.19 ± 5.05	213.15 ± 11.97	0.047
**HbA1C**	6.83 ± 0.14	7.13 ± 0.36	7.65 ± 0.96	0.540	6.87 ± 0.13	7.65 ± 0.96	0.425	6.83 ± 0.14	7.24 ± 0.34	0.323
**Insulin**	41.23 ± 0.27	40.94 ± 0.68	41.57 ± 0.67	0.879	41.17 ± 0.96	41.57 ± 0.67	0.741	41.23 ± 0.27	41.07 ± 0.23	0.637
**HOMA-IR**	2.78 ± 0.08	2.97 ± 0.19	4.04 ± 0.51	0.043	2.80 ± 0.07	4.04 ± 0.51	0.026	2.78 ± 0.08	3.10 ± 0.18	0.042
**HOMA-B**	20.90 ± 0.63	17.92 ± 1.58	11.76 ± 4.23	0.018	20.49 ± 0.59	11.78 ± 4.23	0.016	20.90 ± 0.63	17.17 ± 1.49	0.027

## Discussion

A large number of genetic variants have been investigated for the prediction value of T2DM 13, and they marginally improved prediction beyond noninvasive characteristics in those studies because the accuracy of prediction relies on many factors, such as the number of genes involved, the frequency of risk alleles, and the risks correlated with genotypes 14. The SNP rs174575 of FADS2 gene was selected in the presented study because as per the literature the SNP rs174575 has a combination of two desirable factors (i) It had known linkage disequilibrium (LD) throughout the region, the promoter and intragenic region of FADS2. (ii) It had minor allele frequencies sufficiently prevalent to permit their use in tests of gene-environment and gene-gene interaction 15. Of the three genotype models deduced to study the relationship between genotype and insulin resistance, the recessive model (CC vs CG+GG) was selected based on the number of subjects per group in each model. From the study results it is observed that the HOMA-IR and HOMA-B showed statistically significant difference in the recessive model. The current study findings showed that HOMA-IR was higher in the minor allele carriers when compared to wild type allele and the difference was statistically significant whereas the HOMA-β was lower in the minor allele carriers. Similar results were observed by Kim et al., 2011 in Korean normoglycemic men 16. According to Steer et al., the desatuarse enzyme activity varies between the genotype, thereby altering PUFA metabolism 15. This is of importance because PUFA serve as biological ligands to PPAR-γ (Peroxisome proliferator-activated receptors-gamma) is closely associated with IR (Insulin resistance), Mets (Metabolic Syndrome), and DM (Diabetes mellitus)^17^–^19^. Based on this a suggestive mechanism for the role of rs174575 in T2DM can be derived from this study as depicted in figure 1 (a) and (b). Studies have shown that major homozygotes C have normal PUFA metabolism^13^ because of normal desaturase and elongase activity and produced more omega 3 FAs and anti-inflammatory bioactive mediators, such as leukotrienes and prostaglandins, which act as biological ligands for PPAR-γ. Nevertheless, biological ligands are preferred by PPAR-γ when compared to chemical ligands. Moreover, PUFA blocks NF-kappa B (Nuclear factor Kappa B) reducing inflammation. Together, all factors may increase insulin sensitivity, thereby decreasing insulin resistance Figure 1 (a).

**Figure 1 (a):**
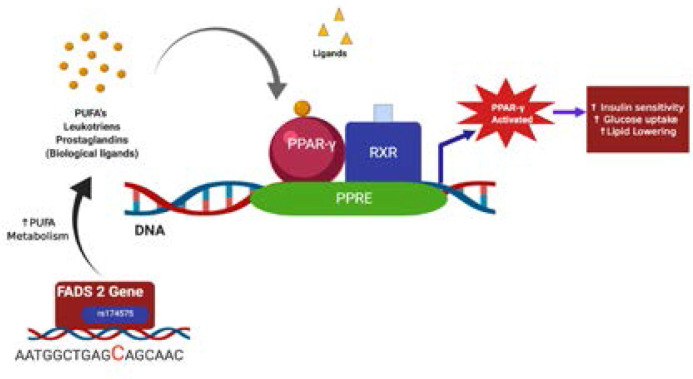
Suggestive mechanism of action: rs174575, CC homozygotes interaction with PPAR-γ as derived from the study (created using Biorender.com)

**Figure 1(b):**
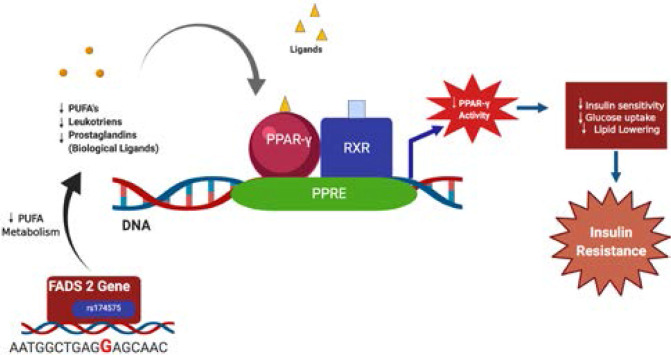
Suggestive mechanism of action of - rs174575-G allele carriers and insulin resistance via interaction with PPAR-γ as derived from the study

Whereas in the minor G allele carriers of rs174575, a PUFA metabolism is altered due to altered desaturase gene expression^13^. Reduced PUFA levels, mainly omega 3 FAs, increased LA (from dietary major sources, such as seed oils), and AA (Arachidonic acid) accumulation activates NF-Kappa B, transcribing COX (Cyclooxygenase) and lipo-oxygenase genes and increasing inflammation. This reduces insulin sensitivity and increases insulin resistance as shown in figure 1 (b).

Therefore, this complex interplay between insulin resistance and gene variants needs to be better understood and elucidated at a molecular level.

## Conclusion

The distribution of rs174575 genotypes differed significantly between the subjects and controls in the present study, and we suggest that having a minor allele genotype alone may significantly increase the risk of incidence T2DM. The study revealed that genetic variation in FADS2 could be an additional facet to consider while studying the risk factors of T2DM.
